# Measles Outbreaks and Implications for Patients Receiving Stem Cell or Cellular Therapies in Canada: Cell Therapy Transplant Canada (CTTC) Infectious Diseases Working Committee

**DOI:** 10.3390/curroncol32090525

**Published:** 2025-09-19

**Authors:** Simon F. Dufresne, Mohammadreza R. Shahmirzadi, Uday Deotare, Dima Kabbani, Shahid Husain, Coleman Rotstein, Seyed M. Hosseini-Moghaddam

**Affiliations:** 1 Division of Infectious Diseases and Medical Microbiology, Department of Medicine, Maisonneuve-Rosemont Hospital, University of Montreal, Montreal, QC H1T 2M4, Canada; sf.dufresne@umontreal.ca; 2 Division of Infectious Diseases, Department of Medicine, London Health Sciences Centre, Western University, London, ON N6A 3K7, Canada; 3 Division of Haematology, Department of Medicine, London Health Sciences Centre, Western University, London, ON N6A 3K7, Canada; 4 Division of Infectious Diseases, Department of Medicine, University of Aberta, Edmonton, AB T6G 2R3, Canada; 5 Ajmera Transplant Centre, Division of Infectious Diseases, Department of Medicine, University Health Network, University of Toronto, Toronto, ON M5S 1A1, Canada

**Keywords:** measles, MMR vaccine, live-vaccine, cell therapy, stem cell transplantation

## Abstract

Measles exposures have historically been rare since the introduction of routine vaccination programs, resulting in a lack of attention from cancer patients, hematopoietic stem cell transplant (HCT) recipients, patients receiving cellular therapy (CT) and their healthcare providers. It is essential to acknowledge the importance of vigilance in these situations. Measles herd immunity has declined significantly in North America due to rising vaccine hesitancy, resulting in outbreaks. Measles can result in severe outcomes, and its reemergence has raised alarm among patients and healthcare professionals caring for HCT/CT recipients. Patients with severe immunocompromising conditions cannot receive live-attenuated vaccines, such as the measles vaccine. The lack of data on measles prevention in this vulnerable group presents significant clinical challenges. In response, Cell Therapy Transplant Canada (CTTC) Infectious Diseases Working Committee has developed a set of frequently asked questions to provide expert guidance to HCT and CT recipients, acknowledging the limited evidence base.

## Simple Summary

Measles is a highly contagious viral infection that can cause serious complications in people with weakened immune systems, including those who have received a stem cell transplant or cellular therapy. In Canada, there is currently an unprecedented resurgence of measles, despite the disease having been eliminated in 1998. Vaccination remains the most effective way to prevent infection, but it may not be recommended for some patients after stem cell transplant or cell therapy. During this resurgence, many patients and healthcare providers are seeking clear guidance on how best to prevent and manage measles in this vulnerable population. In response to this need, the organization Cell Therapy Transplant Canada has asked its Infectious Diseases Working Committee to develop a formal statement. This document is presented in a frequently asked ques-tions (FAQ) format and outlines current best practices.

**Purpose and Scope**

Measles, an RNA virus belonging to the *Paramyxoviridae* family, is a highly contagious pathogen that causes an acute febrile illness [1].While immunity acquired through natural infection—particularly among older adults—and comprehensive vaccination programs have eliminated endemic measles transmission in Canada, periodic localized outbreaks continue to occur [2,3]. These resurgences raise concern within the medical community and the general population, particularly among vulnerable groups such as immunocompromised individuals [4]. Hematopoietic cell transplant (HCT) recipients and individuals who have undergone cell therapy (CT; mainly BMCA- and CD19-CAR T-cell) are particularly at risk, as preventive strategies typically rely on live-attenuated vaccines, which may be contraindicated in certain immunocompromised states [4,5,6].

In 2025, Canada is experiencing its most significant measles resurgence in decades, in keeping with a worldwide increase of cases [2,3]. Amid this ongoing public health challenge, patients, their caregivers, and healthcare providers are seeking clear guidance on the optimal management of measles following HCT or CT. It is essential to emphasize that management strategies should be individualized, with shared decision-making guided by a careful assessment of risks and expected benefits. Key considerations include local epidemiological trends, the degree of immune reconstitution, prior immunization status, and other patient-specific factors [4].

The purpose of this statement and review is to outline the principal elements to consider when managing measles infection in this population. While recognizing that this topic is extensively addressed in existing national and provincial guidelines, the intent of this statement is to provide an overview of the most critical points to support informed decision-making for both patients and healthcare professionals. This document will also direct readers to more comprehensive resources for detailed clinical guidance. Major documents produced by the Public Health Agency of Canada are assembled in Table 1 with hyperlinks to facilitate access. In addition, this document targets the general HCT/CT population and may not address specific considerations related to pediatric patients.

In this report, we address frequently asked questions (FAQs) based on the limited published data regarding measles in oncology and hematopoietic cell transplant (HCT) populations, as well as evidence gleaned from similar immunocompromised groups. Due to the lack of direct evidence, these recommendations also rely on expert opinion, supported by the Cell Therapy Transplant Canada (CTTC).

## 1. FAQ1. What Is the Risk of Acquiring Measles (Natural Disease) in Canada?

Measles was considered eliminated in Canada in 1998, and there has been no endemic transmission of the disease in the country since that time [2,7,8]. The global resurgence of measles and the increased population movement contribute to the ongoing reintroduction of the virus [1,9]. In post-eradication countries such as Canada, cases are typically imported by travelers returning from regions with active transmission, after which local outbreaks may occur among susceptible individuals—primarily those who are unvaccinated [2,7,8]. Despite measles being extremely contagious, outbreaks usually die out due to high levels of herd immunity and public health interventions. As a result, widespread person-to-person transmission across the country is unlikely in the near future. However, larger-scale and more prolonged outbreaks may occur in communities with susceptible hosts and suboptimal vaccination coverage [2,3,10]. The current multijurisdictional outbreak, which began in October 2024 and involves the D8 genotype, also stemmed from an imported case [3].

In 2025, measles cases in the United States and Canada have already reached unprecedented levels since the elimination of endemic transmission in 1998 [2,11]. As of 5 August 2025, Canada reported 4394 confirmed and probable measles cases from 1 January to 26 July 2025 [3]. While incidence remains modest at a population level, this marks a significant surge compared to the 177 cases recorded in 2024 [2,3]. Notably, incidence is not evenly distributed across the country. In 2025, cases were reported in almost all provinces and territories, except Newfoundland and Labrador, Nunavut, and Yukon, but the majority were diagnosed in Ontario (53%) and Alberta (36%) [3]. Age distribution data reveal that 69% of reported cases occurred in children and adolescents, whereas individuals over 55 (i.e., those born before 1970) represented only 1% of cases. Finally, also in 2025, 8% of cases have been noted among vaccinated individuals, and only 5% occurred in persons having received two or more vaccine doses [3]. While this may be less relevant for immunocompromised individuals, it underscores the importance of vaccinating their close contacts to provide indirect protection (see below).

## 2. FAQ2. Is the Risk of Acquiring Measles Higher After HCT/CT?

It is not clear whether receiving HCT or CT increases the risk of exposure to the measles virus [4]. While many immunocompromised individuals tend to limit their social interactions and avoid crowded places, patients who have undergone HCT or CT may be at a higher risk of exposure to the virus in healthcare settings due to the frequency of their medical visits.

On the other hand, HCT or CT recipients are generally considered at higher risk of developing clinical disease upon exposure, due to their impaired immunity [4,12]. The degree of susceptibility is influenced by numerous factors, such as the time elapsed since transplantation, the mode of acquisition of pre-transplantation immunity (vaccine vs. natural disease), the use of active immunosuppression, and the presence of graft-versus-host disease—all of which are considered when tailoring certain interventions, such as post-exposure prophylaxis (see below).

Detection of serum anti-measles antibodies is a commonly used method for objectively assessing measles immunity and estimating susceptibility. Recent data show that approximately 35–70% of stem cell recipients maintain detectable levels of antibodies after transplantation (before MMR revaccination) [13,14,15,16,17,18,19,20,21]. Similarly, unpublished local data from Maisonneuve-Rosemont Hospital (Montreal, Québec) indicate that 67.5% of measles serologies are positive among allogeneic HCT recipients prior to revaccination.

Immunity seems to decline after transplantation, as exemplified in a study conducted at Amsterdam University of Medical Cancer from 2010 to 2017 evaluating measles-specific IgG levels in 91 patients before and after allogeneic hematopoietic stem cell transplantation (HCT). Before HCT, 91% of patients had protective measles IgG levels. However, this figure decreased to 86% at three months post-transplant and further dropped to 61% at 12 months post-transplant [22]. A gradual loss of immunity was also observed by other investigators. Bogeholz et al. [20] additionally found that immunity acquired through natural disease was a strong predictor of positive serology [20]. Graft-versus-host-disease (GVHD) has also been associated with loss of immunity, whereas sex, age (among non-pediatric cohorts), conditioning regimen, and graft source have not been shown to influence post-transplant measles serology [16,20].

Importantly, while serology provides measurable data that help elucidate the dynamics of immunity after HCT and CT, its correlation with clinical protection remains poorly defined [23], and recommendations on how to incorporate this variable into clinical decision-making should be made and interpreted with caution [4,5].

## 3. FAQ3. Is Measles (Natural Disease) More Severe Among HCT/CT Recipients?

Data on the severity of natural measles infections, especially among recipients of HCT or CT, is limited. A measles outbreak in South Korea from 2000 to 2001 involved 16 allogeneic HCT recipients, resulting in at least one fatal case of measles pneumonia in a recipient 15 months following transplantation [24]. During a measles outbreak among pediatric hematology patients, including HCT recipients, in China, more than half of the reported cases were complicated, and over 20% of affected individuals died from the infection [25]. Severe disease cases, including fatalities, have also been reported in individuals with various forms of immunosuppression, such as those undergoing liver or renal transplantation and oncologic patients [25,26,27,28].

In immunocompromised individuals, the clinical spectrum of measles can include severe complications such as pneumonitis, hepatitis, and encephalitis [26,29]. Pulmonary infection may present as measles-associated giant cell pneumonia—also known as Hecht’s pneumonia—or as bacterial superinfection, which was observed in nearly a third of severe pneumonitis cases in a large case series [26,29,30]. Encephalitis displays a broad spectrum of clinical forms, ranging from acute to subacute [26,29,31,32]. Epilepsia partialis continua is a rare form of measles encephalitis encountered only in immunocompromised patients, characterized by focal status epilepticus presenting weeks to months after primary infection [29,33]. Subacute sclerosing panencephalitis is thought to be more common in the context of human immunodeficiency virus infection [34] but is not associated with cellular therapy or transplantation. Of note, measles-induced immune deficiency—a temporary phenomenon known as ‘immune amnesia’—is not well characterized in individuals who are already immunocompromised [1].

Finally, it is essential to note that clinical presentations of measles may be atypical in people with impaired immunity. For instance, the absence of a rash, typically considered a hallmark of measles, is well-documented among patients with acquired-immunodeficiency syndrome and other immunocompromised hosts [29,30,33,35,36]. A very mild rash was also noted is some patients during an outbreak among HCT recipients in Brazil [17].

## 4. FAQ4. What Are the Strategies to Prevent Measles in HCT/CT Recipients?

Preventing measles in recipients of HCT and CT is challenging due to their immunocompromising condition and the contraindications associated with live vaccines. However, several evidence-based strategies and expert-recommended practices can help reduce the risk of measles [4,6,12,37,38,39]. Implementing these strategies can effectively manage the risk of measles in this vulnerable population. These strategies are compiled in Table 2 and further discussed in detail in the following sections.

## 5. FAQ5. How Does a HCT or CT Recipient Reduce the Risk of Exposure?

The measles virus is a highly contagious pathogen, primarily transmitted through respiratory routes. Infection can occur when individuals inhale air from environments where a contagious person has been present or through direct contact with respiratory secretions, such as mucus or saliva, from an infected individual [40,41]. Exposure to the virus may occur when individuals come into contact with surfaces or objects that are contaminated and subsequently touch their eyes, nose, or mouth with unwashed hands. Importantly, the measles virus retains its viability in the ambient air or on surfaces for up to two hours following the departure of an infected individual from the area [42]. Individuals infected with measles can transmit the virus within a time window of four days before the onset of the rash to four days after its appearance. During outbreaks, patients should wear a well-fitting mask in public spaces and prioritize well-ventilated environments, especially in high-traffic indoor areas. Importantly, only N95 respirators have been shown to provide adequate protection against measles [43]. They should also limit exposure to crowded public places, healthcare facilities, and regions with known measles transmission.

Additionally, it is generally recommended that non-immune caregivers and family members of individuals who have received a transplant or cellular therapy be vaccinated against measles. Of note, contrary to live Poliovirus vaccine and smallpox vaccine, the risk of secondary transmission of the vaccine strain from vaccinated contacts to an immunocompromised host is considered negligible, and no special precautions should be followed. Only specific symptoms such as post-vaccination fever and rash would mandate avoiding close contact with a vulnerable host [4,6,44,45].

## 6. FAQ6. Does the Donor’s Immune Status Influence the Recipient’s Measles Risk?

Vaccination of donors can enhance the post-transplant immunity of HCT/CT recipients. When donors are vaccinated against specific pathogens, recipients demonstrate improved immune responses to those organisms and viral antigens after the transplant [46]. However, there is no data supporting this specifically for measles. A study showed that the humoral immunity from the donor did not significantly influence the measles serostatus of recipients after transplantation. In an analysis of antibody levels against measles in 331 patients who underwent allogeneic hematopoietic stem cell transplantation (HCT), the authors concluded that most of the measles antibodies came from remaining host cells rather than being transferred from the donor [20].

Nevertheless, allogeneic HCT donors are still encouraged to be up to date on immunizations, including measles vaccination, if they are non-immune. However, measles vaccination should be avoided within 4 weeks of stem cell harvest [6].

## 7. FAQ7. Does Pre-Transplant Immunity and Vaccination Reduce the Risk of Measles After Stem Cell Transplant and Cellular Therapy?

Recent data from North America indicate that approximately 75% of patients with cancer possess measles immunity, as measured by serology. However, the lowest seroprevalence rates are observed among individuals with hematologic malignancies (63%), those with a history of transplantation or cellular therapy (46%), and individuals aged 30 to 59 years (49–63%). These findings highlight the suboptimal levels of measles immunity among candidates for transplantation or cellular therapy [47].

Whether individuals with evidence of pre-transplant immunity are at lower risk of post-transplant measles remains poorly defined. As outlined in FAQ 2, the degree of post-transplant protection is influenced by numerous factors, including pre-transplant immunity, as well as transplant-related variables that affect immune reconstitution—such as time elapsed since transplantation and the presence of GVHD [16,20]. Overall, it is difficult to isolate the protective effect specifically conferred by the recipient’s pre-transplant immunity. However, naturally acquired immunity (through prior measles infection) is likely an important contributor.

In current clinical practice, pre-transplant immunity alone is generally considered insufficient to ensure protection. Accordingly, careful evaluation for post-transplant revaccination is recommended for all recipients [4,6]. In addition, in the event of measles exposure, pre-transplant immunity data is not typically considered when assessing the need for IVIG or vaccination [4,5].

## 8. FAQ8. What Steps Should Patients Take After Potential Exposure to a Measles Case?

In the event of exposure—defined as being in the same space as a suspected or confirmed measles case for at least 2 h—HCT and CT recipients should be evaluated promptly by their medical team, and post-exposure prophylaxis (PEP) should be offered as early as possible, and no later than six days following the exposure. Immunoglobulins and measles-containing vaccines are both considered effective PEP strategies [48]; however, they are mutually exclusive and should not be administered in combination. The preferred option depends on the individual’s immune status and the time elapsed since exposure. For all early recipients (within 12 months of receiving autologous HCT or CAR T, and within 24 months of receiving allogeneic HCT) and for those with GVHD, PEP consists of immunoglobulins, regardless of immunity or serological status. For patients weighing ≥30 kg, intravenous immunoglobulin (IVIG) should be administered at a dose of 400 mg/kg. For those weighing ≤30 kg, intramuscular immunoglobulin (IMIG) at a dose of 0.5 mL/kg is recommended [5]. Of note, evidence suggests that donor-derived plasma products contain lower levels of neutralizing antibodies following the introduction of routine vaccination programs. Fortunately, recent data from the vaccination era confirm the efficacy of this strategy [48]. When administering immunoglobulin products, caregivers should consider that subsequent serologic assays may yield false-positive results. Therefore, any such testing relevant to immediate or near-future care should be performed prior to treatment. For individuals eligible for vaccination, the MMR vaccine is the PEP of choice and should be administered within 6 days of exposure, although this strategy is most effective when given within 72 h. Patients who have already completed post-transplant measles vaccination may be considered not susceptible—and therefore not in need of PEP—provided that positive measles IgG has been documented. If not, rapid serology may be considered to guide the need for vaccination; otherwise, immediate re-vaccination is recommended [5]. Finally, it is of critical importance that appropriate infection control measures be implemented when caring for exposed patients, who are considered potentially contagious for up to 21 days after exposure [49,50].

## 9. FAQ9. How Do We Diagnose Measles in HCT and CT Recipients?

Healthcare providers should be vigilant for measles in patients presenting with fever, rash, coryza, conjunctivitis, and other compatible symptoms—particularly in those without a history of measles vaccination or prior infection [1]. Additional factors to consider include recent exposure to an individual with a fever and rash or residing in or traveling to an area experiencing a measles outbreak [51]. Clinicians must maintain a high index of suspicion, recognizing that measles may present atypically in immunocompromised individuals—such as with pneumonitis or encephalitis in the absence of the characteristic rash (see FAQ 3). Patients with known or suspected measles should be placed under airborne precautions immediately upon identification. These measures are critical to preventing further transmission, especially in healthcare settings [49,50,52]. Prompt reporting to the local health department is essential, in accordance with public health guidelines.

For the initial diagnosis of measles, we recommend measuring measles-specific serology (IgM and IgG) and detecting measles virus RNA through real-time reverse transcription polymerase chain reaction (RT-PCR) from upper respiratory specimens (throat or nasopharyngeal swab) [2,40].

### 9.1. Serologic Testing

Detection of measles-specific IgM or IgG antibodies may help confirm a diagnosis, but serology comes with significant caveats—especially in immunocompromised hosts—and should not be relied upon as the sole diagnostic method. IgM typically appears 1 to 4 days after rash onset and peaks within the first week; therefore, false-negative results may occur if serum specimens are collected too early. Additionally, immunocompromised individuals may have a delayed or absent IgM response. False-positive results are also possible, and in the context of low disease prevalence—typical of the post-elimination era—this leads to a low positive predictive value. Testing for measles-specific IgG antibodies can help determine immunity status but is less useful for diagnosing acute infection. IgG serology may yield negative results if performed fewer than 10 days after rash onset. For the most reliable results, IgG antibodies should be assessed using paired acute and convalescent sera collected 2 to 3 weeks apart [2,4,40,53].

### 9.2. Nucleic Acid Amplification Tests (NAAT)

RT-PCR is the gold standard for measles diagnosis in immunocompromised patients, including HCT and CT recipients. Nasopharyngeal and oropharyngeal (throat) swabs are the preferred specimens, though urine is also considered an adequate sample. In appropriate clinical contexts, bronchoalveolar lavage fluid and cerebrospinal fluid may also be tested. A key limitation is that viral RNA is typically detectable for a shorter duration than IgM, which may not be the case in immunocompromised hosts [2,4,40,53,54].

## 10. FAQ10. Is There Evidence Showing the Immunogenicity/Effectiveness of the MMR Vaccine After HCT or CT?

Several studies have evaluated the serological response to MMR immunization among HCT recipients, reporting a wide range of efficacy—from 15% to 100%. A number of variables may affect immunogenicity, including graft source, timing after transplantation, age of recipients, and number of vaccine doses [14,16,18,55,56,57,58]. Some of these variables are exemplified in the following studies. At a single institution where pediatric recipients of autologous or allogeneic HCT received early MMR vaccination (beginning at 12 months, with a median of 13 months), seroconversion was observed in 16 of 35 patients (46%). Notably, the seroconversion rate reached 78% among those immunized after 15 months post-transplant, compared to 35% in the subgroup vaccinated earlier (between 12 and 15 months)—suggesting improved immunogenicity with delayed vaccination [55]. In another pediatric cohort of 30 HCT recipients (24 allogeneic), only 4 of 26 (15%) seronegative patients seroconverted after a single dose of MMR vaccine, suggesting that one dose may be insufficient to elicit a proper response in some younger patients [58]. In a mixed population of 23 adult and pediatric cord blood transplant recipients who were seronegative for measles, 15 (65%) seroconverted after receiving one or two doses of MMR vaccine, indicating a comparable response with this stem cell source [14]. Finally, in a report by Machado et al., 73% of patients vaccinated within the past three years were considered immune by serologic testing, compared to only 30% of those vaccinated more than three years earlier—suggesting that vaccine-induced immunity may wane over time [17].

Very limited data are available on the clinical efficacy of measles vaccination in HCT recipients. In a reported Brazilian outbreak involving eight cases within a cohort of HCT recipients, seven of 122 (5.7%) unvaccinated individuals developed measles, compared to only one case among 34 vaccinated patients (2.9%). Notably, the vaccinated individual had received the vaccine three years prior to infection [17]. During another measles outbreak in China, two vaccinated patients (both had received 2 doses) developed measles after transplantation and both survived [25].

## 11. FAQ11. What Complications Are Expected to Occur After Vaccination?

Since MMR is a live-attenuated vaccine, there is a risk of infection with any of the three viruses it contains when administered to immunocompromised patients. Although this risk remains theoretical for mumps, vaccine-strain rubella and measles infections have been rarely reported in immunocompromised individuals, including severe cases with central nervous system involvement and death [59,60,61,62,63,64,65,66]. However, MMR vaccination after HCT or CT is still considered safe when sufficient immune reconstitution has occurred (see FAQ 12) [4,6,37,38]. To our knowledge, well-documented cases of vaccine-associated rubella and measles have been reported only once and twice, respectively, among HCT recipients who received their vaccines after transplantation [59,60,66].

## 12. FAQ12. Is the MMR Vaccine Recommended as a Safe Prevention Strategy in HCT and CT Recipients?

Measles-containing vaccines are considered safe after HCT or CT, provided that certain conditions are met to ensure sufficient immune recovery. The minimal requirements to achieve this threshold are not evidence-based and may vary depending on the source of practice guidelines and the expert opinions behind them. Table 3 summarizes the conditions established by various scientific organizations in their respective practice guidelines.

The current Canadian Immunization Guide recommends measles vaccination 24 months after HCT if the following criteria are met: there is no active chronic graft-versus-host disease (GVHD), no active immunosuppression for at least three months, no relapse of the underlying (immunosuppressive) disease, and the patient is deemed immunocompetent by a transplant specialist. Additionally, live vaccines should be deferred for at least 8 to 11 months following any dose of immunoglobulins. Using serology to guide the need for vaccination (i.e., vaccinating only seronegative individuals) is not recommended by the Canadian Immunization Guide [6].

For patients who have received CAR-T cell therapy, at least 12 months should elapse before considering a measles-containing vaccine. However, no definitive cutoff exists, and a specialist should provide guidance on a case-by-case basis [5,6]. Some experts have proposed a set of parameters—including minimally acceptable immunoglobulin levels, absolute lymphocyte counts, and the absence of significant immunosuppression (e.g., ibrutinib use or >5 mg of prednisone daily) [4].

## 13. FAQ13. Is Early MMR Vaccination Recommended for HCT and CT Recipients in the Context of Outbreaks?

In the context of outbreaks, MMR vaccination has been performed in patients not traditionally considered as having reached complete immune reconstitution as per most accepted criteria. Such occurrences provided occasions to gather important safety data when vaccinating slightly immunocompromised patients [55,66,67,68]. In the largest and most recent experience reported by Desjardins et al., a cohort of 129 HCT recipients—including 54 who underwent allogeneic transplantation—was vaccinated within two years after transplantation in Boston. Only one case of vaccine-associated disease was observed and was confirmed to be caused by the rubella virus [66].

Drawing on these experiences, a guidance framework was proposed by ASTCT to establish safe conditions for early vaccination [4]. It considered parameters such as the level of steroid and immunosuppressive therapy, absolute lymphocyte counts (total, CD4, or CD19), unsupported immunoglobulin levels, and the presence of GVHD.

In line with this statement, we support considering early MMR vaccination for HCT or CT recipients beyond the first year post-transplant or cellular therapy, in the context of community outbreaks with high ongoing transmission. However, we recognize that no clear recommendations can be made regarding safety parameters, and that risks and benefits should be weighed on a case-by-case basis.

## 14. FAQ14. What Are Treatment Options for HCT and CT Patients Infected with Measles?

There is no specific antiviral therapy for measles; management remains primarily supportive. The use of ribavirin, as well as interferon-α or interferon-γ (particularly in cases of measles-associated encephalitis), has been reported in a limited number of case reports and case series, with variable outcomes [32,69,70,71]. We do not recommend antiviral therapy for the management of measles. In addition, while the efficacy of immunoglobulins has been demonstrated when used as post-exposure prophylaxis, there is no evidence supporting their use for the treatment of active infection [1,53]. Vitamin A deficiency is associated with increased disease severity among children with measles, and supplementation has been shown to reduce mortality in low- and middle-income countries. Despite limited data from high-income settings, experts recommend vitamin A supplementation in children, particularly those in high-risk groups such as immunocompromised individuals [1,72]. However, there is no evidence supporting this strategy in the adult population [1].

## Figures and Tables

**Table 1:** Major practical guidelines and reference documents on measles from Public Health Agency of Canada.

Document	Hyperlink
Measles and Rubella Weekly Monitoring Report	https://health-infobase.canada.ca/measles-rubella/ (accessed on 10 August 2025).
Measles: For health professionals	https://www.canada.ca/en/public-health/services/diseases/measles/health-professionals-measles.html (accessed on 10 August 2025)
Immunization of immunocompromised persons, in: Canadian Immunization Guide: Part 3-Vaccination of specific populations	https://www.canada.ca/en/public-health/services/publications/healthy-living/canadian-immunization-guide-part-3-vaccination-specific-populations/page-8-immunization-immunocompromised-persons.html#a19 (accessed on 10 August 2025)
Measles vaccines, in: Canadian Immunization Guide: Part 4-Immunizing agents	https://www.canada.ca/en/public-health/services/publications/healthy-living/canadian-immunization-guide-part-4-active-vaccines/page-12-measles-vaccine.html (accessed on 10 August 2025)
Routine Practices and Additional Precautions for Preventing the Transmission of Infection in Healthcare Settings	https://www.canada.ca/en/public-health/services/publications/diseases-conditions/routine-practices-precautions-healthcare-associated-infections.html (accessed on 10 August 2025)
Updated infection prevention and control recommendations for measles in healthcare settings	https://www.canada.ca/en/public-health/services/diseases/measles/health-professionals-measles/updated-infection-prevention-control-recommendations-healthcare-settings.html (accessed on 10 August 2025)

**Table 2:** Summary of strategies to prevent measles infection after HCT.

Timing	Actions
Before transplantation	○ Evaluating pre-transplant measles immunity through past medical history, vaccination records, and IgG serology testing in some instances. ^1^ ○ Vaccinating non-immune HCT candidates prior to transplantation for those who are not already immunocompromised (e.g., those with metabolic disorders or hemoglobinopathies). ^2^ ○ Vaccinating stem cell donors (see FAQ6). ○ Vaccinating household members and caregivers (see FAQ5).
After transplantation, before exposure	○ Reducing exposure to measles virus (see FAQ5). ○ Vaccinating HCT recipients when considered safe (see FAQ12 & FAQ13).
After transplantation, after exposure	○ Administering post-exposure prophylaxis to susceptible patients. ^3^ (see FAQ8)

Notes: 1—May guide pre-transplant vaccination and help tailor responses to future exposures; 2—provided that the conditioning regimen is scheduled to begin in ≥4 weeks; 3—measles vaccination or immunoglobulins.

**Table 3:** Summary of recommendations for MMR vaccination after allogeneic HCT.

Scientific Organization (Year of Statement or Guide)	Whom to Vaccinate	How to Vaccinate
Canadian Immunization Guide (2025) [6]	○ >24 months after allogeneic HCT ○ No active underlying disease ^1^ ○ No active GVHD ○ No active immunosuppression for >3 months ○ Patient considered immunocompetent by transplant specialist ○ >8–11 months after immunoglobulins	○ 2 doses ○ Serology recommended after 2nd dose
American Society for Transplantation and Cellular Therapy (2019) [4]	○ >24 months after allogeneic HCT ○ No relapse prophylaxis or maintenance therapies ^2^ ○ No immunosuppression ○ No active immunosuppression for >8–11 months, if prolonged therapy was given for chronic GVHD ○ >8–11 months after immunoglobulins	○ 2 doses ○ Serology recommended after 2nd dose in the setting of ongoing transmission
Infectious Diseases Society of America (2013) [37]	○ Only seronegative patients ○ >24 months after HCT ○ No GVHD ○ No active immunosuppression ○ >8–11 months after immunoglobulins	○ 2 doses
International expert consortium ^3^ (2009) [38]	○ Only seronegative adult patients, all pediatric patients ○ 24 months after HCT ○ No GVHD ○ No active immunosuppression	○ 1 or 2 doses ○ Reassess serology after 4–5 years

Notes: 1—Underlying disease for which the transplantation was done, if immunosuppressive; 2—Applicable for hematologic malignancies; 3—Cosponsored by the Center for International Blood and Marrow Transplant Research (CIBMTR), National Marrow Donor Program (NMDP), European Blood and Marrow Transplant Group (EBMT), American Society for Blood and Marrow Transplant (ASBMT), Canadian Blood and Marrow Transplant Group (CBMTG), Infectious Diseases Society of America (IDSA), Society for Healthcare Epidemiology of America (SHEA), Association of Medical Microbiology and Infectious Diseases (AMMI), the Center for Disease Control and Prevention (CDC), and the Health Resources and Services Administration.

## Abbreviations

The following abbreviations are used in this manuscript:

ASTCT	American Society for Transplantation and Cellular Therapy
CAR T	Chimeric antigen receptor T
CT	Cellular therapy
CTTC	Cell Therapy Transplant Canada
FAQ	Frequently asked question
GVHD	Graft-versus-host-disease
HCT	Hematopoietic Cell Transplantation
Ig	Immunoglobulin
IVIG	Intravenous immunoglobulin
MMR	Measles, mumps and rubella
NAAT	Nucleic acid amplification assay
PEP	Post-exposure prophylaxis
RT-PCR	Reverse transcription polymerase chain reaction
